# Holistic health care: Patients' experiences of health care provided by an Advanced Practice Nurse

**DOI:** 10.1111/ijn.12603

**Published:** 2017-10-25

**Authors:** Irene Eriksson, Monica Lindblad, Ulrika Möller, Catharina Gillsjö

**Affiliations:** ^1^ School of Health and Education University of Skövde Skövde Sweden; ^2^ Faculty of Caring Science, Work Life, and Social Welfare University of Borås Borås Sweden; ^3^ Bräcke diakoni Skara Sweden; ^4^ College of Nursing University of Rhode Island Kingston RI USA

**Keywords:** Advanced Practice Nurse, experience, holistic, patient satisfaction, primary health care, qualitative

## Abstract

**Introduction:**

Advanced Practice Nurse (APN) is a fairly new role in the Swedish health care system.

**Aim:**

To describe patients' experiences of health care provided by an APN in primary health care.

**Methods:**

An inductive, descriptive qualitative approach with qualitative open‐ended interviews was chosen to obtain descriptions from 10 participants regarding their experiences of health care provided by an APN. The data were collected during the spring 2012, and a qualitative approach was used for analyze.

**Results:**

The APNs had knowledge and skills to provide safe and secure individual and holistic health care with high quality, and a respectful and flexible approach. The APNs conveyed trust and safety and provided health care that satisfied the patients' needs of accessibility and appropriateness in level of care.

**Conclusion:**

The APNs way of providing health care and promoting health seems beneficial in many ways for the patients. The individual and holistic approach that characterizes the health care provided by the APNs is a key aspect in the prevailing change of health care practice. The transfer of care and the increasing number of older adults, often with a variety of complex health problems, call for development of the new role in this context.

## SUMMARY STATEMENT

What is already known about this topic?

- Advanced Practice Nurse is an established role worldwide but is fairly new in Sweden.
- Patients express a high level of satisfaction with the health care provided by an APN in various settings worldwide.

What this paper adds?

- APNs provide safe and secure individual and holistic health care with high quality.
- APNs have knowledge and skills that they carry out with a respectful and flexible approach.
- APNs convey trust and provide health care that satisfies the patients' needs of accessibility and appropriateness in level of care.

The implications of this paper:

- The transfer of care from hospital to home and the increasing number of older adults, often with complex health problems, call for development of the new role in various contexts in Sweden.
- The individual and holistic approach that characterize APNs needs to be reflected upon in practice, education, and health care policies as key in the prevailing change of health care practice.

## 1 INTRODUCTION

There is an increasing demand in health care systems for advanced health care at an advanced level that also meets expectations of accessibility. This along with a shortage of physicians supports the need to transfer assessments and clinical tasks from physicians to nurses. In order to prepare for this changing role of nurses, education that broadens and deepens knowledge and skills must be provided. This is being done through programs of education for Advanced Practice Nurses in Sweden (Hallman & Gillsjö, 2005; Horrocks, Anderson, & Salisbury, 2002; Laurant et al., 2005, 2008).

In 2003, the University of Skövde was the first university in the Nordic countries to start a program of education for Advanced Practice Nurses (APN) at the Masters level. It was a 2‐year program that met the standards for APN education as set by the International Council of Nurses (ICN) and the Swedish National Board of Health and Welfare. The educational program was collaboratively developed to expand the role and functions of district nurses delivering primary health care in the county of Skaraborg in the western region of Sweden.

Advanced Practice Nurses (APNs), often referred to as Nurse Practitioners (NPs), is a level of practice that was developed in the 1960s in the USA and is now well established in approximately 30 countries all over the world (Andregård & Jangland, 2015; International Council of Nursing Nurse Practitioner/Advanced Practice Network, 2016; Savrin, 2009; Schober, 2006). APNs provide primary, acute, specialty, and long‐term health care to patients over the lifespan with an overall focus on the person as a whole and not only the presenting health problem. APNs have knowledge and skills to make decisions and provide health care autonomously. This includes assessing patients, prescribing and evaluating diagnostic tests, making diagnoses, initiating and managing treatments, prescribing medications, and evaluating the outcomes of their health care. Education of patients is a major component in the APN's role which is focused on informing and guiding patients with decision making and living their lives. The APN's unique emphasis on individually and holistically promoting health and well‐being has resulted in APNs being the preferred health care provider for millions of patients worldwide (Altersved, Zetterlund, Lindblad, & Fagerström, 2011; Bergman, Perhed, Eriksson, Lindblad, & Fagerström, 2013; International Council of Nursing Nurse Practitioner/Advanced Practice Network, 2016; Lindblad, Hallman, Gillsjo, Lindblad, & Fagerstrom, 2010).

An APN is defined by ICN as:

A Nurse Practitioner/Advanced Practice Nurse is a registered nurse who has acquired the expert knowledge base, complex decision‐making skills and clinical competencies for expanded practice, the characteristics of which are shaped by the context and/or country in which s/he is credentialed to practice. A master's degree is recommended for entry level (International Council of Nursing Nurse Practitioner/Advanced Practice Network, 2016).

The new role and function were evaluated in 2006 by Skaraborg Primary Health Care. The evaluation was based on data in interviews with APNs and General Practitioners (GPs) and in questionnaires collected from patients and health care teams. This role was found to be clearly delineated from the role of the GPs and was experienced as an extra resource for the whole health care team. The APN was also viewed as a contributor of continuity and increased accessibility of health care for the patients. However, the participants desired an explicit definition of an APN in the context to clarify the role and its associated responsibilities. Additionally, the lack of an extended right to prescribe medication was highlighted as a limitation in the provision of health care (Altersved et al., 2011; Bergman et al., 2013; Lindblad et al., 2010)

The patients in the study by Bergman et al. (2013) expressed a high level of satisfaction with the health care provided by APNs in primary health care. Several studies report equal (Lenz, Mundinger, Kane, Hopkins, & Lin, 2004; Newhouse et al., 2011; Pinkerton & Bush, 2000) or even higher levels of satisfaction with health care provided by APNs than GPs in various health care settings (Horrocks et al., 2002; Laurant et al., 2005, 2008). The health care provided by an APN has been shown to be more cost effective (Laurant et al., 2005; Newhouse et al., 2011). Reports have indicated equivalence in quality of care and self‐reported perception of health in comparing studies between APNs and physicians. Research has shown that patients' experiences with APNs are supportive and holistic in their approach and provision of health care (Bergman et al., 2013; Laurant et al., 2005, 2008; Wong & Chung, 2006). The acknowledged positive effects of implementing the APN role in primary health care and other health care settings need to be further explored. The aim of this study was to describe patients' experiences of health care provided by an APN in primary health care.

## 2 METHODS

### 2.1 Design

An inductive, descriptive qualitative study design with qualitative interviews was chosen to obtain descriptions from 10 participants who described their experiences of health care provided by an APN in the chosen setting. Face‐to‐face interviews enable immediate confirmation of verbal and non‐verbal expressions in the situation (cf, Kvale, 1983, 1997). A qualitative approach by Graneheim and Lundman (2004) was used to analyze the collected data. This approach for content analysis is interpretive in nature and used to describe or illuminate a phenomenon through identification of manifest (the obvious) and latent (underlying meaning) content in a text.

### 2.2 Sample and data collection

Purposeful sampling was used to recruit participants for the study. Inclusion criteria were that the patient had to be 18 years and above and visit the primary health care center in the western region of Sweden for health problems falling within the scope of APNs' profession. Data were collected during a period of 2 days. Prior to the visit at the primary health care center the patient was triaged by a telephone nurse. Those who fell within the scope of APN profession were listed on the APNs' schedule; however, it was not always clarified for the patient that they were listed to see an APN or a physician. The time set aside for each patient's consultation was equal for physicians and APNs. Patients who visited the 2 APNs during their visiting hours at the primary health care center were asked by the APN after the consultation if they were willing to participate in the study. Thirteen patients were asked to participate, but 3 of them declined. In total, 10 participants were included in the study. The informants' demographics, education, and current health problem are further described in Table 1.

**Table 1:** Characteristics of the study group

Respondent	Civil status	Age	Gender	Type of consultation	Previous visits to the APN competence	Information about the APNs
1	Married	72	Female	Unknown	No	No
2	Single	49	Male	Ear	Yes, once	No
3	Married	63	Female	Sinus	No	Very short
4	Partner	33	Female	Throat	No	No
5	Married	23	Male	Ear	No	No
6	Married	72	Female	Ear	Yes, once	No
7	Married	33	Male	Pneumonia	No	No
8	Single	18	Male	Earwax	No	Yes
9	Partner	27	Female	Ear	No	Yes
10	Married	43	Male	Cold	No	Very short

Qualitative face‐to‐face interviews were conducted to collect data in aim to understand patients' experiences of health care provided by an APN. The interviews were open ended and carried out as a conversation. The opening question was as follows: Would you please describe how you experienced seeing an APN for your health care problem? Subsequent follow‐up questions were used based on the participant's response and orientation in the dialogue to deepen the understanding of the phenomenon. Examples of subject matters for follow up questions were expectations, information and professional approach. The interviews were carried out by the authors (ML, UM) during the spring 2012 in a private setting at the primary health care center. Each interview lasted for approximately 30 minutes and was audio‐recorded and transcribed verbatim.

### 2.3 Rigour

The study was conducted with a focus on the criteria trustworthiness. The concepts of credibility, dependability, and confirmability were used to address this criteria (Lincoln & Guba, 1985). Transparency in collection and analysis of data, and a reflective dialogue approach were used to enhance the credibility. Two of the researchers carried out the interviews which supported the dependability of the study. The findings are illustrated with quotes to preserve confirmability.

### 2.4 Data analysis

The analysis of data started, as consistent with the chosen approach Graneheim and Lundman (2004), a perusal of the text was initially done several times to become familiar with the text and to reflect upon the content. Secondly, the text was read to identify meaning units that described the phenomenon. As a third step, meaning units were condensed, and the essential content was abstracted and labelled with a code. Finally, the codes were compared based on similarities and differences and sorted into preliminary themes. The analysis was an iterative process back and forth across the steps and not a linear process as it might be understood in the description of the analysis. The APNs' professional approach and provision of safe and secure health care with high quality was interpreted as an underlying meaning in the analysis. This manifest content composed the basis for the main theme, “Professional approach that enables safe and secure health care with high quality”. The 3 subthemes could be viewed in light of the main theme. Together with the 3 subthemes, it constituted the coherent whole of the phenomenon. All the authors were involved in all the steps of the analysis of data, and the findings were thoroughly communicated. The authors analyzed the interviews separately, compared, discussed the analysis, and reached consensus about preliminary and final themes which also secured the trustworthiness (cf, Graneheim and Lundman, 2004) in the study.

### 2.5 Ethical considerations

Permission to conduct the study was given by the director at the primary health care center. Oral and written information were given to the 2 APNs and the patients, all of whom gave informed oral and written consent to participate in the study. Approval from the Ethical Review Board was not applied for because it is not needed for this type of study in Sweden. The study was carried out in accordance with the guiding ethical principles; autonomy, beneficence, non‐malicious, and legality in the Declaration of Helsinki (2016). The participants were informed about that the participation was voluntary and confidential and that they could withdraw from the study any time without explanation.

## 3 RESULTS

The analysis of the interviews resulted in 1 main theme and 3 sub‐themes. Quotes are used to illuminate the sub‐themes (Table 2).

**Table 2:** Overview of the results

*Main theme*
Professional approach that enables safe and secure health care with high quality
*Sub themes*
Respectful and flexible approach
Trust in skills and clinical decision‐making
Satisfies need of accessibility of health care on an appropriate level

### 3.1 Main theme

#### 3.1.1 Professional approach that enables safe and secure health care with high quality

The patients experienced that the APNs had knowledge and skills to provide safe and secure health care with high quality. The patients felt that they were treated as individuals with a focus on their whole person and not just their health problem per se. This professional approach was characterized by accuracy, attentiveness, and sensitiveness in the situation which led patients to feeling that APNs could be trusted with their health care problems. The patients were satisfied with the accessibility of health care, and trust was developed despite the fact that the majority of the patients expected to see a physician, because they had not been informed about seeing an APN.

### 3.2 Sub‐themes

#### 3.2.1 Respectful and flexible approach

Patients experienced the APNs as respectful and flexible in their approach to providing health care. This approach was described by the patients as being treated as unique individuals with respect, being genuinely listened to, believed, and confirmed. This contrasted to earlier experiences in which the patients felt a lack of attentiveness because they did not feel listened to or taken seriously in the encounter with the GP. The patients felt that they had to develop *“leather on the nose”*, and be demanding about their needs in order to get attention. The APNs were experienced as being positive and having the ability to seriously listen and confirm the patients' worries and health problems: *“I found it easy to talk to her…just the fact that she listened”*. The APNs' ability to listen and explain was of significance because it contributed to patients feeling that the communications were carried out in a language and on a level that made it easy to understand the given information. This alleviated the patients' distress and helped them calm down. The APNs' professional approach conveyed confidence which developed into trusting relationships. This led to patients feeling comfortable, relaxed, and secure. The APNs were described as polite and flexible in their approach. They focused on the patient as a whole and not only the current health problem. *“One focuses on the patient…are flexible…takes care of and listens to the patient which is important, and also to see what is best for the patient”*. The patients were satisfied with the holistic health care provided by the APN. *“…when one immediately gets the feeling that one can ask for it without any problems, than one is satisfied when one leaves”*. The APNs' respectful and flexible approach was a significant part in the patients' experience of a safe and secure provision of health care with high quality.

#### 3.2.2 Trust in skills and clinical decision‐making

The patients experienced that the APNs conveyed trust and safety by their professional approach. The APN was considered to have knowledge and skills to carry out physical assessments, make clinical judgements, and provide health care that met the patients' needs. The sense of trust was in part developed by the APN's ability to provide exhaustive and detailed information about the patient's diagnosis, and suggested treatment and further investigation and evaluation of the health problem. The patients experienced that the APN took the time to explain the patients' condition, especially when the diagnosis was not as expected. One patient said: *“She explained thoroughly why it wasn't pneumonia”*. Besides the accuracy in provision of health care, the patients valued the APNs' self‐care advices that were focused on promotion of health in regard to individual needs from a holistic perspective. “*She explained very thoroughly. I thought she clearly articulated and explained to me how to do. what?? We got very good information and foremost things to consider in the situation”*.

The patients especially appreciated that APNs in contrast to GPs followed up on their health status and gave feedback even when not expected. A patient said: “*I found it most important that she would evaluate the whole situation. One is not used to that after consulting a physician, it happens very seldom”*. The patients achieved the information that they should contact the primary health care center again if their health status did not improve or if it became worsened. The patients trusted and were content with the health care provided by the APN, which led them to expressing a preference for a consultation with an APN at their next visit. One patient said: *“Same as the last time, I was very content with the results…it all went smoothly the last time so I requested to see an APN again”*. The APNs were trusted and viewed as competent and accurate in their profession which conveyed a sense of confidence and security.

#### 3.2.3 Satisfies need of accessibility of health care on an appropriate level

The patients expressed difficulty in getting appointments with GPs which was described as poor accessibility of health care. If admitted, they often had to spend a long time in the waiting room, and they did not expect anything else. When asked if they wanted to see an APN, they expected the same in regard to waiting time. However, they found that they did not have to wait at all or less time than expected, which they appreciated.

She said that I could get an appointment to the physician but there were 12 patients waiting and she offered an appointment to the APN instead. I said that it is great, I mean perfect. She could do an assessment right away…I expected to be waiting the whole morning but I didn't need to. This was much better.

Furthermore, the patients experienced that the GPs only focused on the patients' actual health problem. By contrast, the APN was described as focusing on the person and the situation individually and holistically. The patients also found the level of care offered by the APN appropriate and it was also easy to talk to her.

Foremost, I find it easier to talk to a nurse instead of a ‘high level doctor’. I also find that a nurse is much more understanding about matters in daily living. A physician can give health care on a specialist level but when it comes to everyday issues, I think that a nurse can deal with this much better.

The patients experienced that the GPs were stressed and rushed through the consultation, and they expressed that they had to be very concrete and succinct. By contrast, the APN was described as less forced and able to take the time needed for the consultation. The visits were described as smooth and relaxed but effective. The contrast in a consultation with a GP and an APN was expressed as:

To compare, every time I visit a GP I get the feeling that I need to rush things since I feel that others are waiting. It is a rather forced situation when one has to shortly explain one's situation. Here it is more relaxed…I find this being a good way of providing health care.

Seeing an APN was experienced as comfortable and relaxed but also effective, fast, and easy. It was a consultation in which the patient felt that enough time was given and that the person was seen as a whole in their specific situation. The APN provided holistic health care that satisfied the patients' needs of accessibility and appropriateness in level of care.

## 4 DISCUSSION

The results in this study showed that the patients were satisfied with the APN's professional holistic approach and the health care provided, which is similar to results in other studies (Agosta, 2009; Bergman et al., 2013; Brown, 2007; Green & Davis, 2005; Schadewaldt, McInnes, Hiller, & Gardner, 2013). Christiansen, Vernon, and Jinks (2013) found that the APNs contribute to improvement of aspects as safety and quality in care, which aligns with the findings in this study. The participants described earlier experiences of poor accessibility to primary health care and long waiting times. They found that the new role APN led to increased accessibility of health care and reduced waiting times which helped to diminish their time away from activities such as work or school, which aligns with earlier research (cf, Agosta, 2009; Fogarty & Cronin, 2008). The participants experienced that the APNs professional approach includes provision of individualized and holistic health care conveyed with a respectful and flexible approach. This individual and holistic approach as a framework for advanced nursing practice can be viewed in light of research (Jennings, Lee, Chao, & Keating, 2009; Wong & Chung, 2006) describing this framework as delineating APNs from GPs in the provision of health care. This framework is acknowledged to be a significant aspect in order to meet the needs of the patients and their families in daily life.

The patients felt confidence in the new profession, despite sometimes not being aware of its existence, and the APNs were trusted with their overall health problems in the situation, (cf, Hayes, 2007; Williams & Jones, 2006). The patients felt more comfortable to talk to the APN about everyday health problems. They did not feel rushed because they experienced that the APN had time and did not convey that they were stressed. This was in contrast with the patients' earlier experiences of seeing GPs. The patients described meetings with GPs that were stressed and did not have the time to fully listen. The results revealed that the patients even preferred to see an APN instead of a GP, which aligns with findings in earlier research (Brown, 2007; Knudtson, 2000). This preference seems to be associated with the APNs' professional approach in which the patient experience being patiently listened to and taken seriously. This approach results in development of trust which in combination with the APN's knowledge and skills facilitate the patient's ability to participate and take responsibility for their own health care (Fagerström, 2011; Green & Davis, 2005).

Similar to earlier research (cf, Horrocks et al, 2002; Laurant et al, 2005, 2008; Martínez‐González et al., 2014), the results in this study indicated that the participants in general are more satisfied with health care provided by an APN than by a GP (cf, Horrocks et al., 2002; Laurant et al., 2005, 2008; Martínez‐González et al., 2014). The acknowledged limitation (Altersved et al., 2011; Bergman et al., 2013; Lindblad et al., 2010) related to the APNs need to consult GPs for prescription of drugs did not seem to influence the participants' level of satisfaction with provided health care. The result in this study supports the earlier noted (Fagerström, 2012; Fogarty & Cronin, 2008) ideal characteristics for an APN, according to the patients, are a professional, individual, and holistic approach to health care.

### 4.1 Limitations

The limitations in this study were the small number of patients and that factors as age, gender, health condition, ethnicity, education, or profession varied because all patients were asked if they were willing to participate in the study. However, this is not a prerequisite in qualitative research. Despite the small number and the variety in age and health conditions, the patients' experiences were similar and consistent across the sample. This can be considered as a surprise because there was a discrepancy in whether or not the patients knew they were seeing an APN or a GP. These aspects, in combination with such factors as face to face interviews, all authors' being involved in the analysis, and the use of quotes to illuminate the themes strengthened the trustworthiness of the study in regard to credibility, dependability, and transferability of the results to other contexts (various settings and age‐groups) (cf, Graneheim & Lundman, 2004; Kvale, 1983, 1997; Stake, 2005).

## 5 CONCLUSIONS

The findings in this study showed that the APNs' way of providing health care and promoting health seem to be beneficial in many ways for the patients. This addresses the need to formalize, develop, and implement the role in various contexts in the Swedish health care system. To date, the limited number of APNs in Sweden is foremost associated with provision of health care in hospitals (Andregård & Jangland, 2015; Jangland et al., 2014) and in primary health care settings (Altersved et al., 2011; Bergman et al., 2013; Hallman & Gillsjö, 2005; Lindblad et al., 2010). However, the increasing number of older adults and the transfer of health care from hospitals to other settings such as the home address the need to implement this new role in community‐based home health care, assisted living facilities, and nursing homes (Smith Higuchi, Hagen, Brown, & Zieber, 2006). The professional, individual, and holistic approach that is acknowledged to characterize the health care provided by the APN is a key aspect in the prevailing change of health care practice. The transfer of care and the increasing number of older adults, often with a variety of complex health problems, call for development of the new role in this context.

## DISCLOSURE

The authors declare no conflicts of interest.

## AUTHOR CONTRIBUTIONS

The authors (ML, UM) collected the data, and all authors (IE, ML, UM, CG) analyzed the data and prepared the manuscript for submission. All authors have read and approved the final manuscript.
